# The Differential Effect of Low-Dose Mixtures of Four Pesticides on the Pea Aphid *Acyrthosiphon pisum*

**DOI:** 10.3390/insects7040053

**Published:** 2016-10-12

**Authors:** Emiliane Taillebois, Steeve H. Thany

**Affiliations:** 1 Laboratoire Stress Oxydant et Pathologies Métaboliques, Institut de Biologie en Santé, Université d’Angers, INSERM 1063, 49933 Angers, France; emiliane_taillebois@yahoo.fr; 2 Laboratoire de Biologie des Ligneux et des Grandes Cultures (LBLGC), Université d’Orléans, UPRES EA 1207-USC INRA, Rue de Chartres, BP 6759, 45067 Orléans, France

**Keywords:** aphid, neonicotinoid, organophosphate, pyrethroid, phenylpyrazol

## Abstract

The modes of action of most insecticides are known, but little information exists regarding the toxicological interactions involving insecticide mixtures at low doses. The effects of mixtures of four insecticides were investigated using LC_10_ values (concentration leading to 10% mortality), acetamiprid (ACE, 0.235 µg/mL), chlorpyriphos (CHL, 107.0 µg/mL), deltamethrin (DEL, 5.831 µg/mL), and fipronil (FIP, 3.775 µg/mL) on the larvae of the pea aphid, *Acyrthosiphon pisum*. After 24 h exposure, 6 of the 11 tested combinations, DEL/FIP, ACE/DEL, CHL/FIP, ACE/DEL/FIP, ACE/CHL/FIP, and ACE/DEL/CHL/FIP, were toxic through an additive effect. Four combinations, ACE/FIP, DEL/CHL, ACE/CHL, and ACE/DEL/CHL had a synergistic effect, whereas only one DEL/CHL/FIP showed an antagonistic effect. The toxic effect of these mixtures was confirmed after 48 h of exposure, revealing an enhanced toxicity of CHL, DEL, and FIP in combination with ACE. We suggest that an insect pest management strategy should be evaluated in the future using different combinations of insecticides.

## 1. Introduction

The use of insecticides remains one of the main pest management strategies in agriculture. Insecticides block physiological processes either directly through action on molecular targets [1,2,3] or indirectly after detoxification mechanisms [4]. Insecticide groups include the IRAC groups, pyrethroids (3A), neonicotinoids (4A), organophosphates (1B), and fiproles (2B) [5]. Pyrethroids like deltamethrin (DEL) kill insects by acting on sodium channels [6], and neonicotinoids like acetamiprid (ACE) act as agonists of the nicotinic acetylcholine receptors [7,8], whereas organophosphates like chlorpyrifos (CHL) inhibit the action of the acetylcholinesterase, resulting in increased acetylcholine concentrations at the synapses [9,10]. More recent compounds like the phenylpyrazol fipronil (FIP) act as agonists of the insect RDL GABA receptors [11,12]. In all cases, insecticides are neurotoxic because they disrupt neurotransmission and alter insect behavior or survival [13].

The development of insect resistance against pesticides and their significant toxicity on the environment lead to new strategies to reduce pesticide use. Indeed, insect resistances are some of the most serious problems in pest control [14,15,16,17,18]. Whole-field applications of pesticides create strong selection pressure on insect populations towards developing resistance. As a result, some companies that experience problems with one compound are likely to switch to using insecticide mixtures to reduce their respective concentrations in the environment in order to delay the appearance of insecticide resistances. Mixtures are combinations of two or more pesticides applied simultaneously. It is expected that their combined effect at low doses may exhibit toxicity through synergistic and additional actions [19,20]. Thus, the toxicity of mixtures is estimated on the assumption that they lead to the combination of substances with independent or similar modes of action. Toxicity can be predicted as follows: additive effects mean that the toxic effect is equal to the sum of the responses to the individual compounds, while synergic effects reflect the situation where mortality is significantly higher than the sum of the individual effects of each compound [21,22]. 

A previous study demonstrated that the neonicotinoid insecticides thiamethoxam (TMX), clothianidin (CLT), and imidacloprid (IMI) had different toxicities against *Acyrthosiphon pisum* larvae [23]. TMX was the most toxic (LC_50_ = 0.259 µg/mL), and CLT was the least toxic (LC_50_ = 3.458 µg/mL) after 24 h of exposure. The toxicity of IMI was intermediate with an LC_50_ value of 0.913 µg/mL. The LC_50_ values were lower after 48 h despite the fact that CLT remained the least toxic (LC_50_ = 0.118 µg/mL), whereas IMI and TMX showed similar effects (LC_50_ values were 0.038 µg/mL and 0.034 µg/mL, respectively) [23]. Recently, by comparing the LC_50_ values of the four compounds CHL, DEL, ACE, and FIP on pea aphid *A. pisum* larvae, we found that the four compounds had different toxicities, resulting in the following toxicity order: ACE > FIP > DEL > CHL [24]. FIP was the most toxic after 24 h of exposure, whereas ACE was the most toxic at 48 h [24]. Interestingly, these insecticides act by different modes of action, and some studies were conducted towards how to best use existing insecticides by combining their different modes of action. Therefore, additional investigations are needed in order to select the most efficient mixtures against insect pests. In the present study, we took advantage of our previous data and evaluated the effects of mixing low doses of CHL, DEL, ACE, and FIP on the pea aphid *A. pisum* larvae. Combined toxicity were observed and compared with a single low dose of each compound.

## 2. Materials and Methods

### 2.1. Insects

The pea aphids, *Acyrthosiphon pisum* strain LSR1 [25], were generously provided by INRA-Rennes IGEPP. Unwinged parthenogenetic females were reared on faba bean (*Vicia fabae*) plants for *A. pisum* in a 16L:8D photoperiod at a constant temperature of 22 °C in a climate chamber. Under these conditions, aphids reproduced via viviparous parthenogenesis as clonal females, and newborn larvae became adults after four molts.

### 2.2. Insecticides

ACE, CHL, DEL, and FIP were purchased from Sigma-Aldrich (Saint-Louis, MO, USA) and dissolved in DMSO (dimethylsulfoxide) to give a final concentration of 50 mg/mL. For intoxication experiments, insecticides were dissolved in an artificial diet at a final concentration of 0.2% DMSO for 100 µg/mL and 2% DMSO for 1000 µg/mL. These concentrations of DMSO were used as controls in insecticidal assays.

### 2.3. Insecticidal Assays

The susceptibility of *A. pisum* to ACE, CHL, DEL, and FIP was determined using an artificial diet bioassay as described in [23,26]. First-instar nymphs were put on a freshly prepared artificial diet system with insecticide (treatment series) or DMSO (control series). For each insecticide, the LC_10_ (concentration leading to 10% mortality) value was tested. The mortality was scored after 24 h and 48 h. Corrected mortality percentages were calculated using Henderson Tilton’s formula after 24 h and 48 h of exposure. The LC_50_ (concentration leading to 50% mortality) of each insecticide tested was previously determined using concentration-response curves [24]. Based on these previous results, we determined the LC_10_ for the four insecticides, and we used these concentrations for insecticidal assays. To explore the combined mixture toxicities, we used equivalent-effect concentration ratios (EECRs) based on the LC_10_ of each insecticide. The concentration ratios (%) of the four pesticides in different EECR mixtures are listed in Table 1. The theoretical mortality was determined as the sum of the individual toxicities and corresponded to a theoretical additive effect of insecticides. The model deviation ratio (MDR) was used to enable a quantitative estimation of the difference in predicted and measured toxicity [27]. MDR was derived by dividing the observed toxicity value by the predicted toxicity. MDR values greater than 1.3 mean that the toxicity of the mixture conformed with synergism, while values lower than 0.7 were taken to conform to antagonism [28].

### 2.4. Statistical Analysis

Statistical analyses were performed using GraphPad Prism 5 (GraphPad Software Inc., La Jolla, CA, USA) with nonlinear regression analysis and *t*-test (*p* < 0.05; *t*-test with Welch’s correction). The theoretical and observed mortality (%) were first compared using a chi^2^-test to determine whether the mixtures had different toxicities compared with the sum of the individual insecticide toxicities.

## 3. Results and Discussion

The pesticide concentrations tested in the current study were determined from concentration-response curves and the resulting LC_10_ values [24]. As a control experiment, we first characterized the percent mortality of each insecticide using the extrapolated LC_10_ values: 0.2345 µg/mL (ACE), 5.831 µg/mL (DEL), 107.0 µg/mL (CHL), and 3.775 µg/mL (FIP). Notably, the LC_10_ value for CHL was much higher than that of the other pesticides in the *A. pisum* LSR1 strain. The percent mortality ranged between 8.61% and 11.30%, which was consistent with theoretical data extrapolated from the toxicological curves [24]. Then, first instar pea aphid *A. pisum* larvae were exposed to the various combinations of ACE, DEL, CHL, and FIP at their LC_10_ values. The percent concentration ratios of the different pesticides were calculated for each mixture as well as the corresponding final insecticide concentration (Table 1). The weak toxicity of CHL induced a very important increase of the global amount of active molecules in the mixture used for toxicological experiments (final insecticide concentration higher than 100 µg/mL). Indeed, when used, CHL represented more than 91% of the active molecules. On the contrary, the mixtures without CHL had a final concentration lower than 10 µg/mL. The results obtained after 24 h of exposure demonstrated that 6 out of the 11 combinations tested led to additive toxicity (Table 2). The binary combinations CHL/FIP and ACE/DEL, when compared to DEL/FIP, increased the mortality but not significantly. The mixture DEL/FIP induced an antagonistic effect, but no significant difference was found with the expected mortality. In a similar way, the ternary combinations ACE/DEL/FIP and ACE/CHL/FIP and the quaternary combination ACE/DEL/CHL/FIP caused additive effects. The binary combinations DEL/CHL and ACE/CHL and one combination including FIP had synergistic effects, as well as the ternary combination ACE/DEL/CHL (Table 2).

Thus, the combination of three insecticides induces synergistic action at low doses. Similar synergistic effects were found in other studies [20,29], as exemplified for the mixtures of destruxins and azadirachtin using the ratios of 8/2 and 1/9 [20], and with a mixture DEL/CHL in the *Spodoptera litura* [30]. The combination DEL/CHL significantly increased the toxicity of DEL in field populations, suggesting that CHL could be used in mixtures to restore DEL susceptibility [30]. Indeed, the mixture DEL/CHL had a synergistic effect on the pea aphid larvae resulting in increased toxicity, while it showed an antagonistic interaction, significantly reducing the percent toxic effect, when associated with FIP. In fact, the toxic effect of the mixture DEL/CHL/FIP was lower than the sum of the three insecticides taken alone, suggesting that the binary combination was synergistic, whereas the ternary combination containing FIP was less effective against *A. pisum* larvae (See Table 2 for all statistical analyses). Similar results were found with the Western flower thrips, *Frankliniella occidentalis Pergande*. Nine binary and three tertiary pesticide mixtures were used in greenhouses. Laboratory results indicated that most of the binary mixtures were synergistic, and only one mixture containing spinosad and bifenazate revealed an antagonistic effect against the Western flower thrips [29].

In our study, the lack of toxicity obtained with the DEL/CHL/FIP mixture could be associated with an antagonistic mechanism between the three compounds. Indeed, antagonistic interactions between pyrethroid and organophosphate were previously reported in different insect species, such as *Culex quinquefasciatus* [31]. We proposed that some insecticide combinations are not effective for a common use against insect pests. On the other hand, our results demonstrated that some mixtures are able to present synergistic or additive effects, thus increasing aphid mortality. Indeed, at 48 h of exposure, we found that some combinations were able to increase the mortality of aphid larvae to almost 100% (Table 3). Five combinations induced a very high percent of mortality: ACE/CHL (83.95%), ACE/DEL/CHL (95.96%), ACE/DEL/FIP (91.93%), ACE/CHL/FIP (92.29%), and ACE/DEL/CHL/FIP (94.42%), demonstrating that these combinations increase the toxic effect of the four insecticides taken separately. Thus, 100% mortality could be expected when insect-susceptible populations are exposed to these mixtures. Two combinations—DEL/CHL (43.13%) and CHL/FIP (44.39%)—showed lower aphid mortality, compatible with the finding that the trend associated with the synergistic and additive effects of these mixtures are not significantly increased between 24 h and 48 h of exposure. This lack of effect could be due to detoxification mechanisms leading to inactive metabolites, as we used LC_10_ values. In this context, the toxicity of CHL is not enhanced by DEL and FIP at 48 h, because they might be degraded or metabolized. According to this hypothesis, the toxicity of DEL, CHL, and FIP at 48 h was enhanced by the addition of ACE. Similar neonicotoinoid insecticides, which act as “super” agonists on insect nicotinic acetylcholine receptors, could have the same effect. It was suggested that combining pyrethroid and organophosphate may result in increased toxicity because organophosphate insecticides bind to the monooxygenase, which blocks the binding and subsequent degradation of pyrethroid insecticides. We proposed that this mechanism is effective for binary combinations compared with ternary combinations with the exception that (1) the insecticide concentrations and ratios are sufficient and (2) considering the resistance mechanisms in the insect species and strains. Mixtures containing the pyrethroid DEL and piperonyl butoxide (PBO) with the neonicotinoids TMX, nitenpyram, and thiacloprid provided higher mortality than DEL and PBO alone, while mixtures containing IMI, CLT, and ACE generated lower toxicity against mosquitoes [32]. Thus, the mixture containing DEL and ACE was less toxic on mosquitoes [32]. Our data confirmed that the DEL/ACE mixture was less toxic compared to the mixture containing ACE, DEL, and CHL (see Table 2 and Table 3). The insecticide combinations tested in the present study could be used in other species as a new strategy against insect pests. However, one should keep in mind that each combination will have to be confirmed, because insecticide efficacy to control particular insect pests will depend on the insect species and strain.

An alternative mechanism to explain synergistic and antagonistic effects could lie in the chemical structure of the compounds. The insecticides ACE, DEL, and CHL were described as highly flexible compared to FIP, which contains a trifluoromethylsulfinyl functional group [24]. We suggest that this structure is relevant for the toxicological effect of the mixture. For example, trifluoromethylsulfinyl group, some conjugation reactions, or any of the FIP metabolites might be involved, resulting in an increase of toxic effects when FIP is added. Nevertheless, insecticide mixtures could be used against insect pests as a relevant strategy to enhance the toxicity of insecticides at low doses.

## 4. Conclusions

In the environment, organisms are exposed to mixtures of chemicals rather than to a single component. Our study demonstrates that, in general, mixtures at low doses of the four common pesticides exhibit additive and synergistic effects. We also found that there were effects using the four pesticides at the LC_10_. Given the critical resistance of insect to insecticides and their complex interactions, we now provide some indications about which combinations could be interesting in the future applications of insect pest management.

## Figures and Tables

**Table 1:** The percent concentration ratios (%) of various pesticide compounds in EECR mixtures. The final insecticide concentration in the different mixture is also reported.

Mixture	Percent Concentration Ratios for Each Insecticide				Final Insecticide Concentration (µg/mL)
	Acetamiprid	Deltamethrin	Chlorpyrifos	Fipronil	
binary combination					
DEL + CHL	0.0	5.2	94.8	0.0	112.8
CHL + FIP	0.0	0.0	96.6	3.4	110.8
DEL + FIP	0.0	60.7	0.0	39.3	9.6
ACE + DEL	3.9	96.1	0.0	0.0	6.1
ACE + FIP	5.8	0.0	0.0	94.2	4.0
ACE + CHL	0.2	0.0	99.8	0.0	107.2
ternary combination					
ACE + DEL + CHL	0.2	5.2	94.6	0.0	113.1
ACE + DEL + FIP	2.4	59.3	0.0	38.4	9.8
ACE + CHL + FIP	0.2	0.0	96.4	3.4	111.0
DEL + CHL + FIP	0.0	5.0	91.8	3.2	116.6
quaternary combination					
ACE + DEL + CHL + FIP	0.2	5.0	91.6	3.2	116.8

**Table 2:** Quantitative estimation between predicted and measured toxicity after 24 h, using chi^2^-test and model deviation ratio (MDR); for each mixture, the deduced type of combined action is reported.

Mixture	Expected Mortality %	Observed Mortality %	χ^2^	*p* (χ^2^)	MDR	Type of Combined Action
DEL + CHL	21.69	32.36	6.70	0.01	1.5	synergistic
CHL + FIP	19.91	24.32	1.22	n.s.	1.2	addition
DEL + FIP	19.00	14.84	1.12	n.s.	0.8	addition
ACE + DEL	20.84	27.08	2.36	n.s.	1.3	addition
ACE + FIP	19.06	28.01	5.19	0.05	1.5	synergistic
ACE + CHL	21.75	31.35	5.42	0.02	1.4	synergistic
ACE + DEL + CHL	32.14	44.45	6.95	0.01	1.4	synergistic
ACE + DEL + FIP	29.45	37.03	2.77	n.s.	1.3	addition
ACE + CHL + FIP	30.36	33.88	0.59	n.s.	1.1	addition
DEL + CHL + FIP	30.30	06.66	26.46	0.001	0.2	antagonism
ACE + DEL + CHL + FIP	40.75	34.74	1.50	n.s.	0.9	addition

χ^2^ = Chi-square value; *p* (χ^2^) = significance of the likelihood test; n.s. = not significant.

**Table 3:** Toxicity of the insecticide mixtures after 48 h in the pea aphid *A. pisum*.

Mixture	Observed Mortality %	χ^2^	*p* (χ^2^)
DEL + CHL	43.13	14.12	0.001
CHL + FIP	44.39	48.48	0.001
DEL + FIP	58.84	2.94	n.s.
ACE + DEL	73.41	745.24	0.001
ACE + FIP	69.76	1512.75	0.001
ACE + CHL	83.95	518.33	0.001
ACE + DEL + CHL	95.96	848.38	0.001
ACE + DEL + FIP	91.93	1206.67	0.001
ACE + CHL + FIP	92.29	1435.43	0.001
DEL + CHL + FIP	18.97	6597.24	0.001
ACE + DEL + CHL + FIP	94.42	2128.33	0.001

χ^2^ = Chi-square value; *p* (χ^2^) = significance of the likelihood test; n.s. = not significant. When expected mortality is higher than 100%, a corrected observed mortality value was used to determine chi-square value.
